# Understanding Sexualized Drug Use: Substances, Reasons, Consequences, and Self-Perceptions among Men Who Have Sex with Other Men in Spain

**DOI:** 10.3390/ijerph20032751

**Published:** 2023-02-03

**Authors:** Daniel Íncera-Fernández, Francisco J. Román, Santiago Moreno-Guillén, Manuel Gámez-Guadix

**Affiliations:** 1 Department of Biological and Health Psychology, Autonomous University of Madrid, 28049 Madrid, Spain; 2 Department of Medicine and Medical Specialties, University of Alcalá de Henares, 28801 Alcalá de Henares, Spain

**Keywords:** sexualized drug use, chemsex, men who have sex with men, sexual minorities, motives, public health

## Abstract

Sexualized drug use (SDU) has been identified as a health risk factor among gay, bisexual, and other men who have sex with men (GBMSM). This study aimed to analyze the associations between SDU frequency and a broad set of substances, motives, consequences, and self-perceptions. Sampling was conducted through an online survey. The final sample consisted of 185 GBMSM aged between 18 and 78 years old (mean age = 38.38, SD = 11.52) who engaged in SDU. We analyzed the frequency of SDU in terms of practicing it “once,” “moderately” (“once a month or less” or “a few times a month”), or “frequently” (from “once a week” to “daily”) during the previous 18 months. A questionnaire was administered through which sociodemographic variables, substances, reasons, consequences, and self-perceptions of SDU practice were analyzed. Participants who did so frequently were significantly more likely to use mephedrone, methamphetamine, and GHB/GBL than those who performed SDU less often (large effect sizes). In addition, habitual SDU was associated with motivations to achieve pleasurable emotions and sensations and manage negative feelings. Health implications, such as blackout moments, were also significantly related to frequent SDUs. Finally, those who practiced frequent SDU perceived it as a severe problem and wanted to control it. These data indicate the importance of raising awareness of chemsex as a public health problem among GBMSM. Specific identification, education, and prevention programs need to be strengthened to reduce the incidence of the most undesirable implications of SDU among GBMSM.

## 1. Introduction

Today, intentional drug use for sex, a phenomenon known as sexualized drug use (SDU), has become a public health priority because of its significant implications for the health of those who engage in it [1]. SDU, which includes the phenomenon of “chemsex” as a particular subset of substances, refers to the intentional use of drugs before or during sexual intercourse to facilitate, enhance, and prolong the sexual experience. It is primarily associated with gay, bisexual, and other men who have sex with men (GBMSM) [2,3]. While some GBMSMs occasionally use drugs for sexual purposes, others do so more frequently [4]. However, not all male sexualized drug users report problematic use [5]. The type of substance, route of administration, and frequency of use could be behind the more adverse outcomes of sexual experience–enhancing drug use [6].

Substances that have been associated with the practice of chemsex include gamma-hydroxybutyrate/gamma-butyrolactone (GHB/GBL), 4-methyl methcathinone (mephedrone), and N-methyl-1-phenyl propan-2-amine (methamphetamine) [7,8]. The use of other, more traditional substances that may be implicated in SDU has also been described, including cocaine, 3,4-methylenedioxymethamphetamine (MDMA), alkyl and butyl nitrites (poppers), ketamine, drugs for improving erection (e.g., Viagra^®^), hashish/marijuana, and alcohol [8,9]. For example, in the study by Vaccher et al. (2020) [10], men who had used poppers, and those who used them more frequently (26.3%), were more likely to report SDU compared to men who had never used them (2.5%). In a study carried out in Spain, Torres et al. (2020) [11] reported that cocaine is the most used substance among men who use drugs before or during sexual intercourse. Some of these substances provide an increase in sexual desire and disinhibition during sexual intercourse [12]. Paradoxically, however, they make it more difficult to achieve or maintain an erection. For this reason, the use of erectile dysfunction drugs to counteract these effects has also been commonly reported [13,14]. In terms of the route of administration, most GBMSM who engage in SDU use oral and/or nasal (inhaled) drugs [2]. Others opt for intravenous administration, a practice that is known as “slam sex” or “slamming” [15], which is more associated with health risks when injecting material is shared [16]. Although the frequency of use is considered among the most important predictors of problematic drug use [17], few studies have analyzed different patterns of SDU frequency. This profile could help identify more significant evidence of vulnerability among people who participate in these sessions.

The reasons that motivate GBMSM to use drugs for sexual purposes have also been key aspects in the growth of SDU. Different studies have described multiple reasons for engaging in the practice. Some users take drugs specifically for fun, to increase euphoria and sexual arousal, and to overcome inhibition and lack of confidence in certain sexual practices [18,19]. Some participants also perceive drug use as an alternative way to socialize, create community networks or emotionally bond with others. In their systematic review, Lafortune et al. (2021) reported that SDU was also for fostering social bonds by sharing intimate spaces or creating a more intense connection with sexual partners. Other SDU participants described it as a means to get out of difficult and painful personal situations or deal with difficult situations in general. For example, Deimel et al. (2016) noted that some GBMSM perceived drug use for sexual purposes as a way to overcome anxieties, insecurities, and difficult life experiences. However, no study has analyzed the reasons for SDU as a function of the intensity of its practice in the Spanish context.

Currently, several studies have found an association between SDU and a decrease in control over possible risks [13,20], which could have significant health implications. Among these implications is a significantly increased risk of intoxication due to the high toxicity of some commonly used drugs in SDU [21] that could increase among people who mix substances [22]. In this sense, Vallecillo et al. (2022) [23] recently concluded that SDU-related intoxication is a growing clinical problem in Spain. In addition, SDU has also been linked to the performance of certain sexual practices (e.g., fisting) and long-term sexual encounters [24]. In their systematic review, Berg et al. (2020) [25] found that some drugs can cause physiological changes in the mucous membranes or blood, increasing susceptibility to sexually transmitted infections (STIs).

Similarly, more intense sexual intercourse for more extended periods of time could be associated with mucosal inflammation and bleeding, increasing the risk of erosions or trauma to the penis or anus [26]. To our knowledge, no study has analyzed a broad set of health implications, including blackout events (mental lapses in memory caused by deficits in memory consolidation due to drug intoxication), according to the frequency of SDU involvement. The specific vulnerabilities of GBMSM to more severe health complications need to be analyzed to develop care and risk management strategies.

Finally, regarding self-perception of SDU, Evers et al. (2020) [27] reported that SDU was related to a positive impact on the lives of 31% of the sexual drug users that were studied, while it was related to a negative effect for 9%. Similarly, Nimbi et al. (2020) noted that those who generally claim to practice SDU describe sessions as positive experiences with negative consequences. Although some GBMSM who practice SDU seem to see themselves as being able to strike a balance between pleasure, safety, and risk, others seem to be concerned about balancing risk with personal safety [6]. This is the case for some SDU participants who had difficulty moderating their drug use [28], sexual activity [29], and sexual self-efficacy [1]. In their study, Bohn et al. (2020) [30] noted that 49.6% of participants who used drugs for sexual purposes reported a loss of control during or after a session in the previous 12 months. For example, Ramos et al. (2021) [31] found that 70% of their participants reported having compulsive sexual behaviors. Considering that SDU is associated with health consequences, health promotion strategies to minimize these complications should be directed towards reinforcing self-control strategies about drug use and self-efficacy skills for sexual practices. Among the critical elements in this self-perception is the frequency of exposure to various risks or harms. However, few studies have focused on analyzing the relationship between SDU and problematic–non-problematic self-assessment and other variables related to self-perception among GBMSM as a function of the frequency of their practice.

Studies on SDU among GBMSM have increased substantially in recent years (e.g., Íncera et al. (2021) [32]. Frequency of use is considered one of the most important predictors of dependent drug use. However, empirical evidence on the link between SDU frequency and substances, reasons, complications, and self-perceptions is still considerably scarce. The primary objective of this study was to analyze the patterns of use of a wide range of substances associated with SDU as a function of their frequency. Many such studies have focused on researching the reasons for performing SDU. However, data on whether these motivations differ depending on the intensity of their practice are limited. The secondary aim of this study, therefore, was to extend previous research on associations between a broad set of reasons for using drugs for sexual purposes and their frequency. Moreover, little is known about the different health implications associated with the frequency of SDU. The third objective of this study was to investigate the relationship between health implications and the degree to which SDU is practiced. Finally, there is a need to understand self-perceptions of the possible risks associated with SDU as a function of its intensity. Therefore, our final objective was to study the association between the frequency of SDU and a broad set of self-perceptions concerning drug use.

## 2. Materials and Methods

### 2.1. Participants

Sampling was done through an online survey. The final sample consisted of 185 GBMSM aged between 18 and 78 years who engaged in SDU (mean age = 38.38, SD = 11.52) residents in Spain. Most self-identified as gay (*n* = 158, 85.4%), while a smaller number identified as bisexual (*n* = 18, 9.7%), pansexual (romantic or sexual attraction to others regardless of gender; *n* = 7, 3.8%), or other sexual orientation (*n* = 2, <2%). Regarding birthplace, 74.6% were born in Spain, and the remaining participants were born in Latin America (15.7), Europe (6.6), North America (1.1%), and Asian countries (1.1%). A majority of those surveyed indicated that they had completed university studies (56.8%). The participants who had not completed university studies at the time of the survey indicated having completed non-university-level technical (20%), high school (16.8%), secondary school (4.9%), no studies/primary school (1.1%), and other studies (0.5%).

### 2.2. Measures

#### 2.2.1. Sociodemographic Questionnaire

The participants were required to provide sociodemographic characteristics as part of the study data. They were asked to indicate their gender identity, age, educational level, monthly income, employment situation, and cohabitation status. 

#### 2.2.2. Sexualized Drug Use Definition

SDU was broadly defined here based on existing evidence. Substances such as poppers [10], alcohol [24], and cocaine [9] have been associated with SDU. Therefore, SDU was defined as the intentional use of drugs (alcohol, cannabis, cocaine, poppers, ecstasy, erectile dysfunction medication, MDMA, GHB/GBL, methamphetamine, mephedrone, ketamine, heroin, benzodiazepine, and other substances) for sexual purposes. Following the methods of Bourne et al. (2015) and Maxwell et al. (2019) to measure SDU, we asked the participants whether they had used substances before or during a sexual encounter to facilitate, intensify, or prolong sexual activity during the previous 18 months. 

#### 2.2.3. Substances for SDU

To explore the potential economic impact of this practice, the participants were asked to report how much money they spent monthly to engage in SDU (<€50, €50–100, €100–200, >€200). We also asked participants who used drugs for sexual purposes to report how often (1 = never, 2 = sometimes, 3 = about half the time, 4 = very often, or 5 = always) they had used each drug in the previous 18 months; these substances included alcohol, hashish/cannabis, cocaine, poppers, ecstasy, erectile dysfunction drugs, MDMA, GHB/GBL, methamphetamine, mephedrone, ketamine, heroin, benzodiazepines, and other substances. We added the category “other substances” to include any drugs not listed in the provided options. Moreover, we asked participants whether alcohol use had led them to use other substances for SDU. Participants who chose intravenous administration as the route of consumption were asked how often they had engaged in slam sex in the previous 18 months (none, once, once a month or less, a few times a month, once a week, more than once a week, or daily) and whether they had shared injection equipment. Finally, the participants were asked to indicate where they usually engaged in SDU (e.g., saunas, sex clubs, private parties, own home, other’s home; see Table 1). 

#### 2.2.4. Reasons for Practicing SDU

The participants were asked to select their motivations for using substances for sexual purposes (e.g., “to escape and/or have a good time,” “to have sex, perform certain sexual practices, or feel that I perform better sexually,” “to feel closer to others and intimate,” etc.). These responses were chosen based on previous research with GBMSM on motivations for engaging in SDU [10,33]. The items are shown in Table 2.

#### 2.2.5. Consequences of SDU Practice

We asked the participants if they had had a blackout event in the previous 18 months, and if so, on how many occasions and with what substances it had happened to them. HIV-positive participants were asked whether they attributed their seroconversion to SDU practice to ascertain some implications of SDU. On the other hand, we asked the participants to indicate if they had had erosions or trauma to the penis or rectum while practicing SDU. For medical care needs during or after SDU, we asked them to indicate if they had received health care for any drug use–related problem; if so, they were asked to indicate what care they had received. These questions were chosen based on previous quantitative studies that examined risky sexual practices associated with SDU [4,34]. All items are shown in Table 3.

#### 2.2.6. Self-Perceptions of SDU

To learn about SDU participants’ perception toward the practice, we offered them different response options (e.g., “It is something that I want to control,” “No problem,” “It is a moderate problem,” “It is a serious problem,” “It is affecting one or more spheres of my life,” “Feeling closer to others and having intimacy”). These responses were developed based on previous research on GBMSM’s self-perceptions of participating in SDU [1,13,35]. All items are shown in Table 4.

### 2.3. Procedure

GBHSM aged 18 years and older were invited to participate through social media, gay dating apps, and information distributed through various LGBTIQ+ associations and NGOs. Inclusion criteria for participation were: (1) being at least 18 years old; (2) being GBMSM; (3) having practiced SDU in the previous 18 months; (4) having resided most of the previous 24 months in Spain. Data were collected anonymously through a self-administered online cross-sectional survey from February to June 2021. The questionnaires were administered to all the participants following the order set out in the previous section. The online survey received 1945 visitors. Of these, 493 participants responded to the questionnaire, and 185 participants who had practiced SDU were selected for this study. The participants were informed that completion of the questionnaire was voluntary, they could choose not to answer the questions, and that participation in the study could be interrupted for any reason without consequences. If they had difficulty answering any of the items, they were encouraged to ask questions via an email created for this purpose. All survey data were collected anonymously. This study followed the ethical standards and guidelines of the Declaration of Helsinki. 

### 2.4. Data Analyses

The final sample (*n* = 185) was divided into three groups according to the frequency of SDU practice over the previous 18 months: once (*n* = 31), moderate (*n* = 71), and frequent (*n* = 83). The moderate group included participants who practiced SDU “once a month or less” or “a few times a month.” The frequent group included participants who performed SDU “once a week,” “weekly,” “more than once a week,” or “daily” over the previous 18 months. The F-statistic was used to compare the groups for continuous variables, while χ^2^ was employed to compare the groups for categorical data. In addition, effect size measures have been included: partial eta squared (proportion of variance that can be partially explained by a given variable in the model after considering the variance explained by other variables in the model) for ANOVA analyses and Cramér’s V (0–0.2, slight association, 0.2–0.6 moderate association, and >0.6 strong association). Note that Fisher’s exact test was conducted when fewer than five participants were in a cell for the chi-squared analysis.

## 3. Results

### 3.1. Sociodemographic Variables Associated with Frequent SDU Practice

First, we analyzed the relationship between the frequency of practicing SDU and demographic variables (Table 1). The data showed that the GBMSM who frequently engaged in SDU were significantly older than those who had done so only once [M = 40.37 and M = 34.52, respectively; F (2,182) = 3.173; p = 0.044; η^2^_p_ = 0.034]. The differences between the frequency of practicing SDU and the demographic variables were not statistically significant.

### 3.2. Substances Associated with Frequent SDU Practice

Those who engaged in SDU frequently were more likely to have used mephedrone, methamphetamine, GHB/GBL, erectile dysfunction drugs, ecstasy, and poppers than those who had done so moderately or once (see Table 1). The effect size of differences were large for GHB/GBL (η^2^_p_ = 0.123), mephedrone (η^2^_p_ = 0.118), methamphetamine (η^2^_p_ = 0.117); medium for drugs for erectile dysfunction (η^2^_p_ = 0.084); and small to medium for ecstasy (η^2^_p_ = 0.049) and poppers (η^2^_p_ = 0.040). Of the participants, 23.7% indicated that drinking alcohol had prompted them to use other substances for SDU, and a quarter (25.5%) had engaged in slam sex over the previous 18 months; of the latter, 12.5% reported sharing injection equipment. More frequently, those who engaged in SDU were more likely to have injected drugs for sexual purposes a few times a month (11.1%; χ^2^ = 24.762; p = 0.016).

**Table 1:** Substances for SDU.

		Total Sample	Frequency of SDU Practice			χ^2^ (*p*; *V*)/*F* (*p*; η^2^_p_)
		*M* (*SD*)/*n* (%)	One Time	Moderate	Frequent	
Monthly money spent at SDU practice (*n* = 179)						
	<€50	58 (32.4)	11 (36.7)	**36 (51.4)**	11 (13.9)	83.027 * **(*p* < 0.001, V = 0.483)**
	€50–100	33 (18.4)	0 (0.0)	9 (12.9)	**24 (30.4)**	
	€100–200	25 (14.0)	0 (0.0)	2 (2.9)	**23 (29.1)**	
	>€200	12 (6.7)	0 (0.0)	1 (1.4)	**11 (13.9)**	
	Nothing; they usually invite me.	51 (28.5)	**19 (63.3)**	22 (31.4)	10 (12.7)	
Substance use in SDU practice (*n* = 185; Likert: 1 = never–5 = always)						
	Alcohol	2.36 (1.37)	2.23 (1.28)	2.48 (1.37)	2.31 (1.42)	0.467 (*p* = 0.628, η^2^_p_ = 0.005)
	Hash/marijuana	2.08 (1.42)	1.90 (1.47)	2.17 (1.41)	2.06 (1.42)	0.384 (*p* = 0.682, η^2^_p_ = 0.004)
	Cocaine	1.75 (1.13)	1.52 (0.93)	1.61 (1.01)	1.96 (1.27)	2.645 (*p* = 0.074, η^2^_p_ = 0.029)
	Poppers	3.20 (1.43)	2.68 (1.40) _c_	3.13 (1.39)	3.48 (1.42) _a_	**3.760 (*p* = 0.025,** **η^2^_p_ = 0.040)**
	Ecstasy	1.75 (1.06)	1.50 (0.82) _c_	1.56 (0.98) _c_	2.01 (1.15) _a,b_	**4.572 (*p* = 0.012,** **η^2^_p_ = 0.049)**
	Viagra/Cialis/Levitra	2.77 (1.57)	1.94 (1.21) _c_	2.64 (1.53)	3.20 (1.60) _a_	**8.198 (*p* < 0.012,** **η^2^_p_ = 0.084)**
	MDMA (M)	1.70 (1.02)	1.43 (0.82)	1.60 (0.94)	1.90 (1.14)	2.897 (*p* = 0.063, η^2^_p_ = 0.031)
	GHB/GLB	2.36 (1.39)	1.61 (1.02) _c_	2.10 (1.13) _c_	2.86 (1.52) _a,b_	**12.479 (*p* < 0.001,** **η^2^_p_ = 0.123)**
	Methamphetamine	1.98 (1.41)	1.58 (1.20) _c_	1.54 (0.95) _c_	2.53 (1.64) _a,b_	**11.989 (*p* < 0.001,** **η^2^_p_ = 0.118)**
	Mephedrone	2.30 (1.49)	1.61 (1.20) _c_	1.96 (1.16) _c_	2.84 (1.64) _a,b_	**11.864 (*p* < 0.001,** **η^2^_p_ = 0.117)**
	Ketamine	1.61 (0.97)	1.48 (0.95)	1.51 (0.91)	1.75 (1.03)	1.525 (*p* = 0.221, η^2^_p_ = 0.017)
	Heroin	1.04 (0.32)	1.00 (0.00)	1.01 (0.12)	1.08 (0.47)	0.918 (*p* = 0.401, η^2^_p_ = 0.010)
	Benzodiazepines	1.20 (0.71)	1.27 (0.91)	1.19 (0.72)	1.17 (0.61)	0.186 (*p* = 0.831, η^2^_p_ = 0.002)
	Others	1.34 (0.97)	1.31 (0.97)	1.14 (0.60)	1.53 (1.19)	2.349 (*p* = 0.099, η^2^_p_ = 0.034)
Has drinking alcohol prompted you to use other substances for SDU? (answer = yes; *n* = 177)		42 (23.7)	8 (27.6)	18 (25.7)	16 (20.5)	0.929 * (*p* = 0.669, V = 0.069)
Frequency of slam sex (*n* = 181)						
	None	134 (74.0)	23 (76.7)	**60 (85.7)**	51 (63.0)	21.621 * (*p* = 0.012, V = 0.262)
	One time	16 (8.8)	6 (20.0)	4 (5.7)	6 (7.4)	
	Once a month or less	16 (8.8)	1 (3.3)	5 (7.1)	10 (12.3)	
	A few times a month	10 (5.5)	0 (0.0)	1 (1.4)	**9 (11.1)**	
	Once a week	1 (0.6)	0 (0.0)	0 (0.0)	1 (1.2)	
	More than once a week	3 (1.7)	0 (0.0)	0 (0.0)	3 (3.7)	
	Daily	1 (0.6)	0 (0.0)	0 (0.0)	1 (1.2)	
Have you shared injection material? (*n* = 48; answer = yes)		6 (12.5)	0 (0.0)	1 (9.1)	5 (17.2)	1.257 * (*p* = 0.695, V = 0.197)
Places where SDU is usually practiced						
	Saunas	41 (21.2)	4 (12.9)	17 (23.9)	20 (24.1)	1.851 (*p* = 0.396, V = 0.100)
	Pubs/nightclubs	20 (10.8)	5 (16.1)	8 (11.3)	7 (8.4)	1.411 (*p* = 0.494, V = 0.087)
	Playful events	8 (4.3)	2 (6.5)	3 (4.2)	3 (3.6)	0.761 * (*p* = 0.798, V = 0.049)
	Sex clubs	40 (21.6)	8 (25.8)	16 (22.5)	16 (19.3)	0.625 (*p* = 0.732, V = 0.058)
	Cruising areas	24 (13.0)	6 (19.4)	8 (11.3)	10 (12.0)	1.364 (*p* = 0.506, V = 0.086)
	Street	2 (1.1)	0 (0.0)	2 (2.8)	0 (0.0)	2.353 * (*p* = 0.304, V = 0.132)
	Private parties	55 (29.7)	8 (25.8)	21 (29.6)	26 (31.3)	0.310 * (*p* = 0.848, V = 0.042)
	Own house	112 (60.5)	13 (41.9)	41 (57.7)	**58 (69.9)**	**7.754 (*p* = 0.021, V = 205)**
	Other’s house	134 (72.4)	23 (74.4)	52 (73.2)	59 (71.1)	0.147 (*p* = 0.929, V = 0.028)
	Others	4 (2.2)	0 (0.0)	0 (0.0)	4 (4.8)	3.634 * (*p* = 0.171, V = 0.165)

Note: Bold values = Frequency higher than expected with standardized residuals >1.96. Underlined values = Frequency lower than expected with standardized residuals < −1.96. For continuous values, Bonferroni differences are shown using subindices: “a” one time, “b” moderate (once a month or less, a few times a month), and “c” frequent (once a week, more than once a week) daily). (*) Fisher’s exact test.

### 3.3. Reasons Associated with Frequent SDU Practice

Next, we analyzed the reasons for favoring the practice of SDU. The results are shown in Table 2. The main reasons for participating in SDU shown in our study were “to evade reality and/or have fun” (52.4%), “to have sex, perform certain sexual practices, or feel like I’m performing better sexually” (47.6%), “to be uninhibited and feel less ashamed” (38.9%), and “for the feeling that you must take advantage of the time and have fun” (33.5%). Of GBMSM, those who engaged in SDU frequently were more likely to report reasons such as “to feel closer to others and be intimate” (31.3%; Fisher’s exact test = 8.797, *p* = 0.012, V = 0.215) and “to isolate me from the world and feel that nothing affects me” (23.5%; Fisher’s exact test = 13.237, *p* = 0.001, V = 0.274), compared with those who engage in SDU in smaller measure. The rest of the reasons did not present statistically significant differences.

**Table 2:** The Reasons and Moments/Situations Favored for Practicing SDU.

	Total Sample *n* (%)	Frequency SDU Practice			χ^2^ (*p, V*)
		One Time	Moderate	Frequently	
Reason for SDU practices					
To evade reality and/or have fun	97 (52.4)	11 (35.5)	36 (50.7)	50 (60.2)	5.685 (*p* = 0.058, V = 0.175)
To be uninhibited and feel less ashamed	72 (38.9)	12 (38.7)	21 (29.6)	39 (47.0)	4.880 (*p* = 0.087, V = 0.162)
To have sex, perform certain sexual practices, or feel like I’m performing better sexually	88 (47.6)	13 (41.9)	29 (40.8)	46 (55.4)	3.734 (*p* = 0.155, V = 0.142)
To feel closer to others and be intimate	42 (22.7)	2 (6.5)	14 (19.7)	**26 (31.3)**	8.797 * (*p* = 0.012, V = 0.215)
For work, since I am a sex worker	1 (0.5)	0 (0.0)	1 (1.4)	0 (0.0)	1.796 * (*p* = 0.551, V = 0.093)
To avoid unpleasant feelings, such as sadness, anxiety, or emptiness	22 (11.9)	1 (3.2)	6 (8.5)	15 (18.1)	5.592 * (*p* = 0.061, V = 0.181)
To not feel alone and isolated	18 (9.7)	1 (3.2)	5 (7.0)	12 (14.5)	3.687 * (*p* = 0.153, V = 0.150)
To isolate myself from the world and feel that nothing affects me	27 (14.6)	2 (6.5)	4 (5.6)	**21 (23.5)**	13.237 * (*p* = 0.001, V = 0.274)
It helps me to make sure that everything does not matter to me	36 (19.5)	4 (12.9)	11 (15.5)	21 (25.3)	3.119 * (*p* = 0.211, V = 0.135)
For the feeling that you have to take advantage of the time and have fun	62 (33.5)	12 (38.7)	19 (26.8)	31 (37.3)	2.377 (*p* = 0.305, V = 0.113)
None of the above	12 (6.5)	4 (12.9)	6 (8.5)	2 (2.4)	5.084 * (*p* = 0.062, V = 0.162)

Note: Bold values = Frequency higher than expected with standardized residuals >1.96. Underlined values = Frequency lower than expected with standardized residuals < −1.96. (*) Fisher’s exact test.

### 3.4. Consequences Associated with Frequent SDU Practice

Table 3 shows some of the possible undesirable consequences of SDU. The data showed that 18.5% of the participants reported some blackout moment. Specifically, those who engaged in SDU frequently (32.9%) were significantly more likely to have had a blackout moment than those who engaged once (0.0%) or moderately (9.9%) (Fisher’s exact test = 22.194, p < 0.001, V = 0.342). GBMSM who reported practicing SDU more frequently were significantly more likely to have had a blackout moment when using GHB/GLB and, to a lesser extent, mephedrone or ecstasy. Although the differences were not statistically significant, 21.8% of the HIV-positive participants attributed their seroconversion to frequent SDU. In addition, more than a third of frequent sexual drug users (35.9%) had had erosions or trauma to the penis or rectum during their sessions. Finally, we analyzed whether the SDU participants required medical assistance. Up to 12% of GBMSM who habitually practiced SDU over the previous 18 months reported needing health care while using drugs for sexual purposes.

**Table 3:** Consequences of SDU Practices—Blackout Event, HIV, Erosions, and Health Assistance.

		Total Sample	Frequency SDU			χ^2^ (*p,* V)/*F* (*p*; η^2^_p_)
		*M* (SD)/*n* (%)	One Time	Moderate	Frequent	
Have you experienced a blackout event? (answer = yes)		34 (18.5)	0 (0.0)	7 (9.9)	**27 (32.9)**	22.194 * (*p* < 0.001, V = 0.342)
Number of blackout vents		5.32 (1.97)	0 (0.0)	4.57 (0.79)	5.54 (1.97)	1.325 (*p* = 0.259, η^2^_p_ = 0.044)
Drug associated with blackout events (*n* = 34; answer = yes)						
	Hash/Marijuana	6 (17.6)	0 (0.0)	3 (4.2)	3 (3.6)	0.911 * (*p* = 0.741, V = 0.084)
	Cocaine	5 (14.7)	0 (0.0)	0 (0.0)	5 (6.0)	4.857 * (*p* = 0.071, V = 0.185)
	Poppers	4 (11.8)	0 (0.0)	2 (2.8)	2 (2.4)	0.582 * (*p* = 0.999, V = 0.068)
	Ecstasy	6 (17.6)	0 (0.0)	0 (0.0)	**6 (7.2)**	6.176 * (*p* = 0.022, V = 0.203)
	MDMA (M)	4 (11.8)	0 (0.0)	0 (0.0)	**4 (4.8)**	3.634 * (*p* = 0.172, V = 0.165)
	GHB/GLB	22 (64.7)	0 (0.0)	3 (4.2)	**19 (22.9)**	17.417 * (*p* < 0.001, V = 0.310)
	Methamphetamine	5 (14.7)	0 (0.0)	1 (1.4)	4 (4.8)	1.852 * (*p* = 0.394, V = 0.121)
	Mephedrone	9 (26.5)	0 (0.0)	1 (1.4)	**8 (9.6)**	6.221 * (*p* = 0.032, V = 0.201)
	Ketamine	5 (14.7)	0 (0.0)	0 (0.0)	5 (6.0)	4.857 * (*p* = 0.071, V = 0.185)
	Heroin	1 (2.9)	0 (0.0)	0 (0.0)	1 (1.2)	1.484 * (*p* = 0.999, V = 0.082)
	Benzodiazepines	1 (2.9)	0 (0.0)	0 (0.0)	1 (1.2)	1.484 * (*p* = 0.999, V = 0.082)
	Others	3 (8.8)	0 (0.0)	1 (1.4)	2 (2.4)	0.621 * (*p* = 0.999,V = 0.068)
HIV seroconversion because of SDU practices (answer = yes)		18 (15.0)	1 (6.3)	5 (10.2)	12 (21.8)	3.353 * (*p* = 0.211, V = 0.179)
Erosions or trauma to the penis or rectum from SDU (answer = yes)		48 (27.6)	6 (21.4)	14 (20.6)	**28 (35.9)**	4.896 (*p* = 0.086,V = 0.168)
Health assistance because of SDU (answer = yes)		14 (7.6)	1 (3.3)	3 (4.2)	**10 (12.0)**	3.639 * (*p* = 0.185, V = 0.152)
Type of health assistance (answer = yes)						
	Hospital admission	7 (3.8)	0 (0.0)	1 (1.4)	**6 (7.2)**	3.837 * (*p* = 0.103, V = 0.165)
	Home care	2 (13.3)	0 (0.0)	0 (0.0)	**2 (2.4)**	1.725 * (*p* = 0.655, V = 0.116)
	Outpatient care	4 (26.7)	0 (0.0)	0 (0.0)	**4 (4.8)**	3.634 * (*p* = 0.166, V = 0.165)
	Emergency visit	9 (69.0)	1 (3.2)	1 (1.4)	**7 (8.4)**	3.856 * (*p* = 0.105, V = 0.152)
	Others	2 (20.0)	0 (0.0)	1 (1.4)	1 (1.2)	0.626 * (*p* = 0.999, V = 0.048)

Note: Bold values = Frequency higher than expected with standardized residuals > 1.96. Underlined values = Frequency lower than expected with standardized residuals < −1.96. (*) Fisher’s exact test.

### 3.5. Self-Perceptions Associated with Frequent SDU Practice

We analyzed the participants’ self-perceptions of SDU, and the results are presented in Table 4. As shown, 36.8% of the participants perceived it to be “a way to enjoy oneself, meet people, feel good, and have a good time,” a third (33.0%) did not perceive it to be a problem, and 29.2% indicated that “it is something that I want to control.” Comparing GBMSM who practiced SDU frequently with those who practiced it moderately, the former were more likely to report it to be a serious problem (16.9% and 2.8%, respectively; Fisher’s exact test = 8.498, p = 0.009, V = 0.211), “it is something that I want to control” (43.4% and 15.5%, respectively; χ^2^ = 15.178; p = 0.001), and “it is affecting one or more spheres of my life” (21.7% and 1.4%, respectively, Fisher’s exact test = 17.038, p < 0.001, V = 0.299). Finally, GBMSM who reported moderate frequency of SDU practice were more likely to report that SDU was not a problem than those who frequently practiced it (49.3% and 15.7%, respectively; Fisher’s exact test = 20.939, p < 0.001, V = 0.336). In addition, we analyzed the intention to exercise some control over SDU. The results showed that more than half of the participants (61.3%) had tried to reduce or stop using drugs for sexual purposes at some time.

**Table 4:** Self-Perceptions of SDU.

		Total Sample	Frequency SDU			χ^2^ (*p, V*)
		*n* (%)	One Time	Moderate	Frequent	
Self-perceptions of SDU (answer = yes)						
	It is a slight problem	43 (23.2)	8 (25.8)	21 (29.6)	14 (16.9)	3.602 (*p* = 0.165, V = 0.140)
	It is a moderate problem	28 (15.1)	1 (3.2)	7 (9.9)	**20 (24.1)**	9.807 * (*p* = 0.007, V = 0.234)
	It is a serious problem	19 (10.3)	3 (9.7)	2 (2.8)	**14 (16.9)**	8.498 * (*p* = 0.009, V = 0.211)
	It is a personal mistake and a failure that will not happen again	11 (5.9)	5 (16.1)	2 (2.8)	4 (4.8)	5.864 * (*p* = 0.036, V = 0.197)
	It is something that I want to control	54 (29.2)	7 (22.6)	11 (15.5)	**36 (43.4)**	**15.178 (*p* = 0.001, V = 0.286)**
	No problem	61 (33.0)	13 (41.9)	**35 (49.3)**	13 (15.7)	**20.939 (*p* < 0.001, V = 0.336)**
	I am aware that it is a problem, but I am not taking action to fix it	16 (8.6)	1 (3.2)	3 (4.2)	**12 (14.5)**	5.695 * (*p* = 0.044, V = 0.187)
	It is affecting one or more spheres of my life	21 (11.4)	2 (6.5)	1 (1.4)	**18 (21.7)**	**17.038 (*p* < 0.001, V = 0.299)**
	It is a way to enjoy myself, meet people, feel good, and have a good time	68 (36.8)	7 (22.6)	25 (35.2)	36 (43.4)	4.316 (*p* = 0.116, V = 0.153)
Have you ever tried to reduce or end your SDU practices? (answer = yes)		106 (61.3)	14 (51.9)	39 (51.1)	53 (66.6)	1.977 (*p* = 0.372, V = 0.107)

Note: Bold values = Frequency higher than expected with standardized residuals > 1.96. Underlined values = Frequency lower than expected with standardized residuals < −1.96. (*) Fisher’s exact test.

## 4. Discussion

SDU has become an individual and collective challenge with significant health implications. The present study focused on analyzing a wide set of factors associated with the frequency of SDU among GBMSM, including the substances, reasons, consequences, and self-perceptions for participating in it. The results of this study suggest that those who engage more frequently in SDU: (1) have a higher prevalence of using GHB/GBL, mephedrone, and methamphetamine, (2) practice slam sex a few times each month, (3) do so because they feel closer to others and have greater intimacy, (4) have had a blackout event when they use GHB, and (5) perceive of SDU as a problem. These results extend previous research by showing numerous differences between those who engage in SDU frequently and those who do so less often. The implications of these results are discussed below.

In terms of substances used, we found that GBMSM, who are frequently involved in SDU, present a higher consumption of chemsex substances (methamphetamine, mephedrone, GHB/GBL), erectile dysfunction drugs, ecstasy, and poppers. In this regard, the effect sizes were large for chemsex substances and smaller for erectile dysfunction drugs, ecstasy, and poppers. These data suggest that chemsex substances best differentiate the frequency with which SDU is practiced. Furthermore, individuals who engage in SDU frequently choose substances such as poppers and erectile dysfunction medications [8,36]. The easy access and lower risk perception of these drugs, as well as the need for their users to counteract the effects caused by the consumption of some other substances, could partly explain these findings. Along these lines, chemsex substance use has been associated with increased sexual arousal, but paradoxically, it may also make it more difficult to achieve or maintain an erection [37]. These findings suggest that some substances are used more frequently among GBMSH who engage in SDU, increasing the likelihood of abuse and dependence even for substances with a lower risk profile. Thus, efforts to identify those with dependent substance use need to be intensified to minimize the harms and risks associated with SDU. 

We know that people who participate in SDU are more likely to engage in risky sexual practices, such as slam sex [16]. However, this is one of the few studies that has analyzed the intravenous use of substances for sexual purposes associated with SDU frequency. We found that those who engaged in SDU more frequently were more likely to participate in slam sex a few times per month. These data suggest that more acute SDU may be related to higher-risk sexual practices. Interventions with users who engage in SDU should focus efforts on reinforcing specific interventions based on risk minimization among those who choose to use the intravenous route of administration for sexual purposes.

The reasons for engaging in SDU are important when focusing on preventive and care interventions targeting GBMSM who do so. We found that those who were more frequently involved in SDU were more likely to report feeling closer to and having more intimate moments with other men and isolating themselves from the world, and being unaffected by anything than those who were moderately involved in SDU or involved only once. This may be an indication that the reasons for participating in SDU are diverse and not mutually exclusive, and they depend largely on individual differences. Thus, SDU may become, for some people, a means to achieve pleasant emotions and sensations, as well as a maladaptive strategy to manage negative feelings. These results extend previous qualitative research, whereby SDU has been related to social and psychological factors such as enhancement of sexual experience, development of greater connection with sexual partners [1,38], and avoidance of unpleasant feelings and emotions [39]. These data reinforce the idea of promoting an approach focused on the individual needs of sexual drug users incorporating approaches both focused on the psychological or social implications derived from SDU and the promotion of alternative leisure strategies. In addition, these results suggest the need for further research aimed at addressing the role of factors associated with stress in sexual minorities (internalized homophobia, victimization experiences, and perceived stress) and their relationship with SDU. 

To our knowledge, this is the first study to analyze differences in the frequency of SDU practice across a broad set of health implications, including blackout events. Our study shows that those who practiced SDU more frequently were more likely to have a blackout event, especially when they ingested GHB. In this sense, chronic GHB consumption could involve significant health complications due to its narrow margin of safety. This is probably related to the fact that a higher frequency of consumption and not knowing the exact concentration and amount of product being used could contribute to GHB poisoning. In this regard, previous studies have found that people who ingest GHB are more likely to experience serious adverse effects such as seizures, respiratory depression, hypothermia, coma, and death [40,41]. Another possible explanation for these findings is that polysubstance use among people who practice SDU more frequently could be related to more serious health consequences. Previous research has indicated that polydrug use has been associated with more negative consequences for physical and mental health [42,43]. These data indicate the importance of raising awareness of SDU as a public health problem among GBMSM. The use of potentially dangerous substances, such as GHB/GBL, the detection of new substances, and the combination of substances represent the most significant challenges facing SDU studies and intervention futures. 

We also found that those who practice SDU more frequently perceive it as a problem that they want to control and that it affects their lives to a greater extent than those who practice it less regularly. Similarly, our data also indicate that men who practice SDU moderately are more likely to report that it is not a problem for them. More acute use may be perceived as problematic, whereas moderate use may be perceived as low risk. This could be due to two possible hypotheses. First, habitual SDU use can limit the ability to control consumption. In this sense, the effect of some drugs on control and risk minimization could contribute to greater difficulty in moderating drug use [6] and, therefore, favor a worse self-perception of SDU. Second, previous problems in inhibitory control can lead to more acute drug use for sexual purposes. This refers to the ability to inhibit dominant or automatic reactions [44]. In this sense, difficulties in controlling consumption can increase vulnerability to compulsive drug use and, therefore, its problematic self-perception.

This study has several limitations that should be pointed out. The first concerns the cross-sectional nature of the data, which means that temporal relationships between variables cannot be established. Future longitudinal studies should explore the temporal order between health implications and SDU. Second, our sample was limited to a group of GBMSM who use drugs for sexual purposes in Spain and is not representative of the groups of men who practice SDU in different countries; thus, caution is recommended when generalizing the results. In this regard, future studies should replicate and extend the results described here using larger samples of GBMSM and those in different territories. Third, the period prior to the evaluation coincided with some of the lockdowns and restrictions due to SARS-CoV-2, which considerably limited social contact. For this reason, we decided to consider the period spanning the previous 18 months with the aim of more accurately identifying sexualized drug users. Longer periods, however, can be subject to recall bias, distorting the accuracy of the information over time. Fourth, it is necessary to consider that the findings of this study should be interpreted in an epidemiological context in which there was a risk of SARS-CoV-2 infection, which could have biased, at least in part, who participated in the study and how respondents answered the survey questions. In addition, the present study took an exploratory approach to analyze the reasons in favor of practicing SDU, which led to the inclusion of options that were too broad or ambiguous (e.g., “to evade reality and/or have fun”). The reasons associated with frequent SDU practice should be more exhaustively explored in future studies. In addition, the individual questions and dichotomic variables used in the present study might have low reliability. Future studies should progress toward the design and validation of specific questionnaires in the field of SDU with adequate psychometric properties. Future studies should also include additional assessment strategies, such as in-depth interviews. Moreover, the multiple comparisons carried out in this study could increase the rate of type 1 errors. Finally, this study only measured factors related to physical health. Future studies should include additional indicators, such as inhibitory control and impulsivity. 

## 5. Conclusions

Substance use for sexual purposes is a public health problem affecting GBMSM. This study is one of the few conducted to date that has analyzed a set of factors associated with SDU as a function of the intensity of its practice. The results suggest that regular SDU is significantly related to substances with a high-risk profile, such as methamphetamine, mephedrone, and GHB/GBL. In addition, motives for engaging in SDU relate to attaining pleasurable sensations and/or managing negative feelings. SDU is also associated with health implications and worse self-perception among those who practice it more frequently. These data highlight the need to reinforce care for people who engage in SDU more frequently and who could present dependent consumption. This emphasizes the need to implement screening programs in systems aimed at the early detection of health issues related to SDU. This strategy entails the development of training plans aimed at helping direct care professionals carry out screening and subsequent referral to the most appropriate resource based on users’ individual needs. Moreover, it is necessary to implement specific interventions designed to provide information on possible risks and how to manage them effectively and to offer health and care resources to people involved in SDU.

## Data Availability

Not applicable.
